# Clinical Decision Making and Outcome in Routine Care for People with Severe Mental Illness (CEDAR): Study protocol

**DOI:** 10.1186/1471-244X-10-90

**Published:** 2010-11-10

**Authors:** Bernd Puschner, Sabine Steffen, Mike Slade, Helena Kaliniecka, Mario Maj, Andrea Fiorillo, Povl Munk-Jørgensen, Jens Ivar Larsen, Anikó Égerházi, Zoltan Nemes, Wulf Rössler, Wolfram Kawohl, Thomas Becker

**Affiliations:** 1 Department of Psychiatry and Psychotherapy II, Ulm University, Ludwig-Heilmeyer-Str. 2, 89312 Günzburg, Germany; 2 King's College London, Institute of Psychiatry, Box P029, De Crespigny Park, London SE5 8AF, UK; 3 Department of Psychiatry, University of Naples SUN, Largo Madonna delle Grazie, 80138 Naples, Italy; 4 Unit for Psychiatric Research, Aalborg Psychiatric Hospital, Aarhus University Hospital, Mølleparkvej 10, 9000 Aalborg, Denmark; 5 Medical and Health Science Center, Department of Psychiatry, University of Debrecen, Nagyerdei krt. 98, 4012 Debrecen, Hungary; 6 Department of General and Social Psychiatry, University of Zurich, Militärstrasse 8, 8021 Zurich, Switzerland

## Abstract

**Background:**

A considerable amount of research has been conducted on clinical decision making (CDM) in short-term physical conditions. However, there is a lack of knowledge on CDM and its outcome in long-term illnesses, especially in care for people with severe mental illness.

**Methods/Design:**

The study entitled "Clinical decision making and outcome in routine care for people with severe mental illness" (CEDAR) is carried out in six European countries (Denmark, Germany, Hungary, Italy, Switzerland and UK). First, CEDAR establishes a methodology to assess CDM in people with severe mental illness. Specific instruments are developed (and psychometric properties established) to measure CDM style, key elements of CDM in routine care, as well as CDM involvement and satisfaction from patient and therapist perspectives. Second, these instruments are being put to use in a multi-national prospective observational study (bimonthly assessments during a one-year observation period; N = 560). This study investigates the immediate, short- and long-term effect of CDM on crucial dimensions of clinical outcome (symptom level, quality of life, needs) by taking into account significant variables moderating the relationship between CDM and outcome.

**Discussion:**

The results of this study will make possible to delineate quality indicators of CDM, as well as to specify prime areas for further improvement. Ingredients of best practice in CDM in the routine care for people with severe mental illness will be extracted and recommendations formulated. With its explicit focus on the patient role in CDM, CEDAR will also contribute to strengthening the service user perspective. This project will substantially add to improving the practice of CDM in mental health care across Europe.

**Trial register:**

ISRCTN75841675.

## Background

Severe mental illness (SMI) substantially contributes to disability and global burden of disease. In the general population in Europe of adult age, the prevalence rate (12 months) for severe mental illness is about 2.2% [1] indicating that about 11 million people in the European Union are affected by clinically and socially disabling conditions with a high need for intensive and long-term professional treatment.

While people with SMI in Europe receive professional health care in different treatment settings with the majority being cared for by community-based services, there is a lack of knowledge on clinical decision making (CDM) and its outcome in routine care. This is especially disturbing since during the last decades, mental health research has resulted in a large number of interventions with proven efficacy whose implementation requires communication between patient and clinician as well as actions following their interactions. It is unknown whether, to which extent, and how positive patient outcome following such interventions depends upon patient-clinician interaction or CDM. We argue that the major reason for this lack of knowledge is that research on CDM in health care has primarily focused upon well-defined situations in physical conditions, while there are only very few studies on CDM in routine care for people with SMI with its high demands on patient engagement, ensuring continuity of care and establishing stable therapeutic relationships.

### Contextualising clinical decision making

Given a general increase in interest in patient-centered [2] or patient-focused [3] approaches, it has been suggested that research on clinical decision making should become a priority. E.g. the NIMH *Bridging Science and Practice Report *[4] specifically recommended to encourage "the development of methods to study and incorporate clinician and patient/consumer decision making processes into intervention research" (recommendation #24), as well as "the improvement of methods for both evaluating clinician implementation and patient/consumer adherence to treatment recommendations and estimating the consequences of these variations on the effectiveness of treatment" (recommendation #26).

Since the 1960s, concepts developed within the framework of decision theory have been applied to health care research. Research on CDM has drawn upon several conceptual approaches such as information processing, social judgement theory, and expected utility theory. Until the 1980s, this research almost solely focused on clinician decision making [5]. There is considerable debate on what constitutes a "good" clinical decision. In a well-defined one-time clinical decision scenario, an ideal decision is conceptualised as being the sole responsibility of the physician who decides via a rational process taking into account scientific evidence and clinical experience. In this scenario it is assumed that patients would make the same decision if provided with the same information [6]. Information would be communicated to the patient, but the context of decision making is rather unimportant in such a scenario [7]. CDM viewed this way is a rational and linear process lending itself readily to systematic analysis [8]. However, since CDM rarely takes place in such clear-cut situations, less decontextualised models of decision making have been developed.

**Figure 1:**
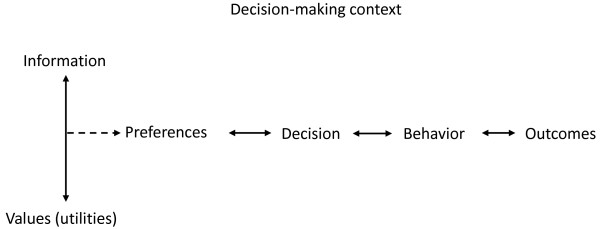
**A simplified model of decision making (adapted from Wills et al. **[5]).

Entwistle et al. [9] proposed a contextualised sequence of activities in decision making consisting of: (a) recognition and clarification of a problem; (b) identification of potential solutions; (c) appraisal of potential solutions; (d) selection of course of action; (e) implementation of the chosen course of action; and (f) evaluation of the solution adopted. Similarly, Rothert et al. [10] developed a general framework (see Figure 1) which conceptualises the decision making process from the patient's perspective, and shows how - via the process of decision making - individual-level variables might be related to individual- and service-level outcomes.

This framework suggests that effective decision making depends on accurate information regarding the risks and benefits as well as the likelihoods of relevant outcomes and an understanding of the values relevant to the decision. Decisions are made after preferences have been formulated by combining information and values and in turn affect patient behaviours (e.g. treatment adherence) and outcomes. *Information *refers to decision-relevant data, e.g. risks and benefits associated with a given treatment, which should be easily accessible and comprehensible. *Values *represent the individual attractiveness of health states taking into account a given treatment's negative aspects, e.g. unwanted side effects. The context of decision making refers to various aspects of a person's everyday life including structure and accessibility of the healthcare system as well as nature, duration and severity of the illness. *Preferences *indicate greater liking of one treatment option compared to another and are conceptualised as an interaction between information and values. *Outcomes *as the result of patient behaviour include patient health status or costs of care [5].

### Types of clinical decision making

Furthermore, increasing attention has been given to patient involvement in CDM. Charles et al. [11] proposed three general types of treatment decision making (see Table 1) in which three main activities (information transfer, deliberation, and deciding about implementing treatment) are being considered. Analogue types taking into account desire for information and for treatment choice have been suggested [12]: (a) Professional choice: The clinician decides and the patient consents; (b) Shared decision making: Information is shared and both decide together; (c) Consumer choice: The clinician informs and the patient makes the decision.

**Table 1:** Models of treatment decision making.

	Paternalistic model	Shared decision making model	Informed (patient) model
Information transfer	One-way (doctor to patient) transfer of minimum medical information necessary for informed consent	Two way: doctor provides all medical information needed for decision making. Patient provides information about preferences	One way (doctor to patient) transfer of all medical information needed for decision making

Deliberation	Doctor alone, or with other doctors	Doctor and patient (possibly with others)	Patient (possibly with others)

Decision about implementing treatment	Doctor	Doctor and patient	Patient

*Note*. Adapted from Entwistle et al. [9].

Coulter [12] argues that different models may be appropriate at different times. While shared decision making has been advocated as a promising approach in order to improve matching of treatments to patients, patient satisfaction, and outcome, the extent of decision making involvement that is necessary to a shared decision making process is under debate. E.g. there is consistent evidence for a high (and often unmet) need for treatment information by patients, but it has also been found that in some instances, patients do not want to be responsible for making treatment decisions.

Thus, in high-stake decisions such as emergency and life-threatening situations, a paternalistic approach might be more reasonable while in situations where treatment decisions are more controversial, the shared decision making or even the consumer choice model might be preferable [13]. There is no evidence on which approach is to be preferred in CDM situations regarding the care of people with SMI.

### Clinical decision making in chronic illness

Much of the literature on CDM has focused on acute and serious medical conditions. In immediate and high-risk acute care situations, the clinician is often seen as the primary source of medical knowledge and as the sole authority for deciding on treatment options, which is most often restricted on whether or not to comply with his or her recommendation [8]. However, decision making in chronic conditions such as SMI differs from decision making in acute care in several aspects. Watt [8] introduced a framework for understanding decision making in chronic conditions which can also be applied to SMI. The author delineated several factors impacting differently upon decision making in acute vs. chronic illness (see Table 2).

**Table 2:** Factors in clinical decision making in acute vs. chronic illness.

Factors	Acute illness	Chronic illness
Nature of illness	Discrete; time-limited; treatable	Pervasive; long-term; manageable

Decisions	Cure focused	Control focused
Nature	Deal with cause; minimal side effects	Symptom reduction; sequellae prevention; side effects trade-off
Number	Single	Multiple; repetitive

Evidence used	Focused on illness	Focused on illness plus lifestyle; little on multiple chronic conditions and their interaction

Decision making relationship	Patient and treatment focused; Permission for provider to act	Consumer and symptom focused; Permission for consumer to act

Decision making environment	Temporary disruption until patient is well	Permanently altered to accommodate symptoms and management

*Note*. Adapted from Watt [8].

According to this framework, CDM in persistent conditions such as SMI - as opposed to well-defined acute care situations - has to take into account that: (a) treatment focus is on long-term disease management; (b) a high number of decisions have to be arrived at frequently, often together with more than one service provider and/or carers; and (c) patients in general are highly knowledgeable about their illness. Due to increased accessibility of treatment-relevant information e.g. via internet of self-help sources, patients might even have more recent and better information than their service providers.

### Research on clinical decision making in general

Research on CDM has focused on a range of physical conditions, predominantly in well-defined short-term life-threatening events (heart attack, stroke), but has also looked at prolonged states of ill health, e.g. cancer and fibromyalgia. The research focus has been primarily on formal decision analysis in high risk-risk acute treatments such as one-time acute treatment choices in surgery via hypothetical scenarios/vignettes. These approaches have been criticised for being overly cognitive and decontexualised, and also for lack of generalisability of results [5].

From this type of research, decision trees and decision aids have been generated. While the number of decision support applications has been increasing rapidly during the last years, there is still a range of open questions regarding their use, content, and format [14]. With the exception of a decision aid for depression medication, there is currently no publicly available decision aid for mental illness [5]. Information for patient decision making varies widely in its quality and comprehensibility and is not always adequately accessible to patients to be useful for decision making [15]. Some positive effects of decision aids have been identified, e.g. on patients' knowledge and understanding of their condition, treatment options, and outcome probabilities, as well as on agreement between patient preferences and subsequent treatment decisions [16]. However, a review of 200 decision aids has also shown decision aids failed to improve satisfaction with decision making, anxiety, and health outcomes [17].

### Research on clinical decision making in mental health

It is doubtful whether the general concepts of CDM described above carry over well to mental health care provision. On one hand, mental health is unique to medicine in that some patients are being treated against their will [13]. Therefore, generic findings on CDM may not be applicable in certain instances, e.g. among people who have experienced involuntary mental health treatment [18]. Also, there might be patients who fail to perceive personal control of choices as a reality [5]. On the other hand, patients are increasingly recognised as key decision makers in mental health care, and it has generally been shown that choice is important to patients and improves engagement with services [13].

Some recent studies report that people with mental illness want a say in their care. Hamann et al. [19] found that in patients with schizophrenia the desire for decision making was slightly stronger than among patients in primary care [7]. Similar results have been reported for community mental health patients in England [20]. The authors also showed that there was a great variation in the extent to which patients wanted to be involved in decisions regarding their care. Furthermore, low levels of patient involvement in medical decisions were observed in primary care consultations of depressive patients [21] while effects of the decision process on patient satisfaction and treatment outcome were not assessed. A recent RCT found that sharing medical decisions with acutely ill people with schizophrenia is feasible. However, effects of a shared decision intervention were only shown with regard to the level of knowledge about the illness (which was higher) and perceived involvement in medical decisions (which was increased), but not for symptom level [22].

A few studies on giving patients a choice in selecting between a limited number of (mostly two) different broad treatment options (e.g. psychotherapy vs. medication) have been conducted. While some positive effects have been shown for treatment adherence (lower drop-out rates for participants who were given a choice) in people with depression [23,24], results regarding clinical outcome are mixed. No clear effects of patient preference on outcome were revealed in studies with cocaine abusers [25] and with people with depression in primary care [26], whereas effects have been shown in people with alcohol abuse [27] and with phobia [28]. It has also been shown via conversation analysis that patients with SMI not only want a say in their care but are actively involved in negotiating care [29].

### Assessment of clinical decision making and of treatment outcome in mental health

In order to scrutinise the relation between quality of CDM and outcome in the care of people with SMI, feasible measures with good psychometric properties including sensitivity to change are necessary to capture vital elements of CDM and treatment outcome.

While some instruments for measuring the quality of decision making in general health care have been put forth [7,30], instrument development for assessing the quality of decision making in mental health conditions has begun only recently [31]. Instruments predominantly focus on patients' appraisal of their involvement in treatment decisions or on patient autonomy. However, there is a lack of instruments capturing crucial basic features of CDM in mental health care including: (a) characteristics of clinical decisions; (b) patient (and clinician) satisfaction with clinical decisions; and (c) actual (vs. preferred) patient involvement in making clinical decisions.

On the other hand, during the last years significant progress has been made in measuring mental health outcomes. First, standardised versions in several European languages of instruments measuring key outcome domains in the treatment of people with severe mental illness have been presented [32]. Second, it has been shown that continuous assessment of treatment outcome via standardised instruments is feasible in people with mental illness [3,33]. Furthermore, recent evidence indicates that people with mental illness are well equipped and able to use modern communication technologies for outcome assessment [34-36], and that reliable and valid outcome ratings can be obtained via the internet [37].

### Research need

While substantial evidence has been accumulated via rather refined and theory-based methods for CDM in physical conditions, research on CDM in mental illness is still at an early stage. By incorporating multiple methods, research on CDM should go beyond the laboratory setting which is also consistent with calls to study mental health phenomena and interventions under less than controlled real-world conditions [38].

Research has focused either on how to help patients make decisions, or on how to understand the degree of involvement in decision making desired by the patient, but not on the nature (kind, number) of CDM in everyday life. Key research challenges in CDM in the care for people with SMI include [5,8]:

• Descriptive research and instrument development focussing on how decisions are actually made in routine care, and how the process of decision making relates to everyday behaviours and outcomes;

• Improvement of measures for characterising decision making processes that are matched to study populations, complexity, and type of decision making, especially in people with severe and long-standing mental disorder who are obliged to make multiple and repetitive treatment decisions, often in cooperation with more than one treatment provider;

• Information about the psychological impact of patient participation in making complex and stressful decisions;

• Decision making styles of both patients and of providers and how these styles are enacted in a variety of CDM encounters;

• How decision making results in congruent or conflicting outcomes and how all participants evaluate such outcomes.

Furthermore, quality of CDM in the care of people with SMI has yet to be studied from an international perspective which would yield insights into commonalities and differences of CDM between different countries and mental health service systems. The most important approach (i.e. both clinically relevant and crucial for clinical governance) would be a focus on what level of participation a patient wants in their care, and whether a good match between desired and experienced level of participation has any impact on either satisfaction or outcome.

### Research question

Main objective of this study is to develop a methodology to assess the scope and quality of clinical decisions in the care of people with SMI from both the patient and clinician perspective, and to specify how and to what degree CDM in routine care affects patient behaviour and short- and long-term treatment outcome. Thus, the main study hypotheses are:

(1) Primary

(a) The quality of CDM can be adequately described by taking into account decision making styles, satisfaction with decision making, and type of decision making ("paternalistic" vs. "shared" vs. "informed") from both patient and clinician perspective as well as their congruence or incongruence.

(b) The type and quality of CDM is positively related to treatment outcome in the routine care of people with SMI.

(c) Actual CDM in routine care depends on context variables, i.e. varies for different types of decision and is susceptible to change over time.

(2) Secondary

(a) The relation between quality of CDM and outcome is affected by a number of covariates at the level of

(i) the patient (sociodemographic status, clinical characteristics, symptom severity),

(ii) the clinician (experience, expertise),

(iii) their interaction,

(iv) the quality of their therapeutic relationship,

(v) the congruence or incongruence of CDM process from patient and clinician perspective,

(vi) the service system (availability of and access to treatment).

(b) The quality of CDM is related to service use, i.e. more adequate service use is to be found in people with a high quality of CDM.

## Methods/Design

**Figure 2:**
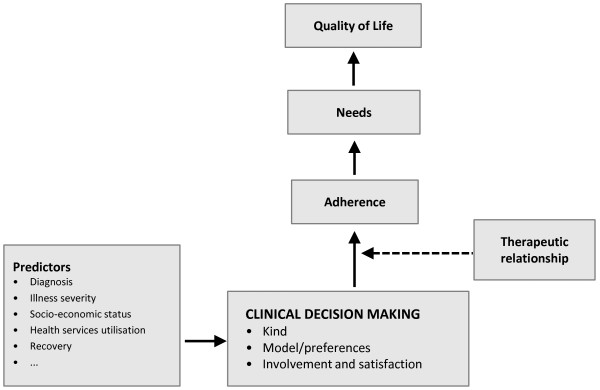
**Model of clinical decision making in the care of people with severe mental illness to be tested in CEDAR**.

The study "Clinical decision making and outcome in routine care for people with severe mental illness" (CEDAR) will test a model of CDM in people with SMI as shown in Figure 2.

This model shows that the focus of CDM is the interaction between patient and clinician who are characterised by a number of attributes including decision making style and form a therapeutic alliance of a certain quality. Both patient and clinician as well as their alliance are affected by aspects of the service system, e.g. whether a given intervention is available or affordable. This builds the context of clinical decisions which during a given period differ in kind (e.g. related to pharmacological or psychosocial treatments) and number, as well as in extent to which they contain elements of "shared decision making". Intermediate consequences of decision making are satisfaction with decision making from the perspective of both patient and clinician and patient behaviour (adherence). The result of CDM is clinical outcome which should capture different domains (symptoms, quality of life, needs) and be observed from different perspectives (patient, clinician, and independent rater). There are a number of possible feedback loops. The most obvious is an outcome-oriented adaptation of clinical decisions following evaluation of adherence to and outcome of the previous decision.

Specifically, as derived from this model, the relationships between the following variables will be investigated in a prospective multi-centre study:

(a) Patient desire for and experience of involvement in CDM;

(b) Congruence of perception of CDM (CDM type, satisfaction) between patient and clinician;

(c) Quality of the therapeutic relationship;

(d) Patient satisfaction with their involvement (the hypothesis being that a high match between desired and experienced involvement will be associated with higher satisfaction);

(e) Treatment adherence (with the same hypothesis);

(f) Outcome (needs, quality of life, symptoms).

### Design and recruitment

*CEDAR *is a naturalistic prospective longitudinal observational study with bimonthly assessments during a 12-month observation period (T0-T6). Participants are being recruited from caseloads of outpatient/community mental health services at six centres throughout Europe: Department of Psychiatry II, Ulm University, Germany (coordinating centre); Section of Recovery at Institute of Psychiatry, London, U.K.; the Department of Psychiatry at Second University of Naples, Italy; the Department of Psychiatry at Debrecen University, Hungary; the Unit for Psychiatric Research at Aalborg Psychiatric Hospital, Denmark; and the Department of General and Social Psychiatry at University of Zurich, Switzerland.

Before the start of recruitment in November 2009, the study protocol has been approved by all centres' ethics committees. Only subjects will be included who provided valid informed consent. Each potential participant in this research project, prior to consent, will be clearly informed of its goals, its possible adverse events and the possibility to refuse to participate or to withdraw consent without any adverse consequences. Informed consent will be asked only of persons able to freely understand and question.

### Inclusion and exclusion criteria

Screening for inclusion and exclusion criteria is carried out by qualified research workers in close contact with clinical staff.

#### Inclusion criteria

• Adult age (18-60 years) at intake;

• Mental disorder of any kind as main diagnosis established by case notes or staff communication using SCID criteria;

• Presence of severe mental illness (Threshold Assessment Grid ≥ 5 points and illness duration ≥ 2 years);

• Expected contact with mental health services (excluding inpatient services) during the time of study participation;

• Sufficient command of the host country's language;

• Capable of giving informed consent.

#### Exclusion criteria

• Main diagnosis of mental retardation, dementia, substance use or organic brain disorder;

• Cognitive impairment severe enough to make it impossible to give meaningful information on study instruments;

• Treatment by forensic psychiatric services.

### Instruments and data collection

Using intensive literature search and focus group methodology [39], three instruments were developed in the course of preparing the start of the study (April - October 2009):

(1) *Clinical Decision Making Style Scale *(CDMS CEDAR) in order to comprehensively assess nature (preferences, autonomy, information seeking) and stability of patients' and clinicians' decision making style both at baseline and at one-year follow-up (21 items);

(2) *Clinical Decision Making in Routine Care Scale *(CDRC CEDAR) in order to measure key aspects of CDM from the patient and clinician perspectives as they unfold in routine care (4 items plus 3 follow-up items measuring degree of implementation of the decision identified at the last *CEDAR *assessment and significant life events since then).

(3) *Clinical Decision Making Involvement and Satisfaction Scale *(CDIS CEDAR) in order to assess subjective satisfaction and involvement with clinical decision making (7 items).

Table 3 lists the instruments used in *CEDAR *to assess relevant variables by time points of their application and rater perspective(s).

**Table 3:** Study instruments by perspective and measurement point.

*Variable*	*Instrument*	*Perspec-tive*	*Measurement point*		
			
			t0	t1-t5	t6
Clinical characteristics (diagnosis, illness duration)	Structured Clinical Interview for DSM-IV on the basis of case notes (SCID [45,46])	P^R^	✓		

Sociodemographic status, service use	Client Sociodemographic and Service Receipt Inventory (CSSRI-EU [49])	P^R^	✓		✓

Illness severity	Threshold Assessment Grid (TAG [47])	P^R^	✓		✓

CDM Style	Clinical Decision Making Style Scale (CDMS CEDAR)	P/S	✓		✓

CDM in Routine Care	Clinical Decision Making in Routine Care Scale (CDRC CEDAR)	P/S	✓	✓	✓

CDM Involvement and Satisfaction	Clinical Decision Making Involvement and Satisfaction Scale (CDIS CEDAR)	P/S	✓	✓	✓

Needs	Camberwell Assessment of Need Short Appraisal Schedule (CANSAS [48,49])	P	✓	✓	✓

Quality of Life	Manchester Short Assessment of Quality of Life (MANSA[50])	P	✓		✓

Therapeutic relationship	Helping Alliance Scale (HAS [51])	P/S	✓	✓	✓

Symptomatic impairment	Outcome Questionnaire (OQ-45.2 [52])	P	✓		✓
	Health of the Nation Outcome Scale (HoNOS [53])	S	✓		✓

Functioning	Global Assessment of Functioning Scale (GAF [54])	S	✓		✓

Recovery	Stages of Recovery Instrument (STORI-30 [55])	P	✓		✓

*Notes*. CDM: Clinical Decision Making; P: Patient; S: Staff; P/S: Patient and Staff; P^R^: Patient, researcher-led, t0: baseline assessment; t1-t5: intermediate assessments (2, 4, 6, 8, and 10 months); t6: final assessment (12 months).

Unit of analysis for the CDM measures is always the decision arrived at during the meeting prior to the current assessment point as indicated by the patient. Furthermore, adherence to the decision indicated at the previous time point is scrutinized via the respective items in the CDRC follow-up ("Have you implemented the decision identified in your last *CEDAR *assessment two months ago?").

All instruments used were made available in all centres' languages via intensive forward and backward translation following common standards [40]. Data is collected via questionnaires (filled in by the patient or his or her key worker) or via interviews conducted by the *CEDAR *study worker. Data entry modes are via computer or paper-pencil forms.

### Sample size

Sample size calculation was performed for the analyses of the primary outcome, i.e. whether needs rated via the CANSAS-P are affected by the quality of decision making during the one-year observation time. Following Hedeker et al. [56], assuming a constant group effect over time with a random-effect structure and auto-correlated residuals, and estimating a panel attrition of 5% at each measurement point, a small effect size (0.2 SD) should be detected with a power of 0.80 at a two-tailed significance level of 0.05 with a group sample size of N = 222 for six time points (and of N = 238 for eight time points). Required sample size for seven time points as in our design was estimated via interpolating the difference in N from six to eight time points with all other elements of the equation remaining unchanged: N = (222 + 238)/2 = 230. For this analyses, participants will be grouped in two categories of quality of decision making, resulting in a total required group size at T0 of N = 460. This means that baseline sample size to be recruited at each centre is N = 77 (after rounding to the next higher integer).

Variation between centres will be taken into account by treating centres as clusters. According to Donner [57], the variance inflation factor (or design effect) is given by *IF *= 1 + (*m *- 1) * *ρ*; where *m *= cluster size, and *ρ *= ICC (inter-cluster correlation).

With m = 77 and ρ = .003, IF = 1,22, resulting in an adjusted sample size of N = 561 (94 per centre).

### Procedures

Descriptive reports include absolute and relative frequencies for categorical variables, and means and standard deviations (and minimum, median and maximum as well the 25%- and 75%-percentiles where applicable) for continuous variables. Between-centre differences will be exploratively tested by χ^2^-Tests for factors and by T-tests or ANOVAs resp. for continuous variables. The effect of the intervention on needs, quality of life and symptomatic impairment will be tested by means of hierarchical linear models [41] with the time variable t (0, 2, 4, 6, 8, 10 and 12 months). Random effects will be observations "within" subject over time, and fixed effects will effects of time, quality of clinical decision making and other covariates (see Figure 2) on the given outcome measure. All available data will be used in the data analysis. Sum scales will be prorated in case of missing values on less than 80% of the single items making up the score. Furthermore, cluster analyses will be used to arrive at meaningful categories of quality of decision making from the CDM measures applied, and chain modelling [42] will be used to draw possible causal inferences from the panel data.

## Discussion

During the last decades, almost all EU member states have undergone substantial psychiatric reforms. In addition, mental health practice and research has provided a large number of pharmacological and psychosocial interventions with proven efficacy and also effectiveness for improving clinical outcome and quality of life among people with SMI. Still, as stated in the EC's recent *Green Paper *[43], "mental health of the EU population can be considerably improved" (p. 3). We believe that an effective way to achieve this is not so much the development of further new interventions, but to see to that existing effective treatment are being offered and utilised via specifying best practices of clinical decision making in the care of people with severe mental illness.

As described above, during the last decades considerable evidence has been accumulated on CDM in physical conditions, especially in well-defined high-risk situations. However, there is a shortage of research findings on CDM in the routine care for people with persistent diseases such as severe mental illness.

High-quality descriptive research is needed in order to gain knowledge on the structure and process of CDM in the routine care of people with SMI. Furthermore, more knowledge is needed on the immediate and long-term effects of CDM on satisfaction with decision making, patient behaviour, and most importantly on treatment outcome. The rigorous scrutiny of these issues in a well-designed large multinational prospective observational study will yield insights into general effective ingredients of CDM and into specific ingredients applicable to specific mental health service systems at individual (patient, clinician) and service level, but also into factors not readily amenable to change, and thus substantially advance the state-of-the-art in the field.

In the following, *CEDAR's *expected impacts will be outlined in relation to the topics of the call (FP7-HEALTH-2007-3.1-4: Improving clinical decision making, [44] p. 44).

### Develop and validate methodology to measure the quality of clinical decisions

Instruments to capture structure, process, and outcome of decision making in the care of people with SMI will be developed and empirically validated: (a) Structure: Clinical decision making style and key elements of clinical decisions; (b) Process: Contribution of patient and clinician to CDM, patient behaviour (immediate and long-term); (c) Outcome: Satisfaction with CDM, and clinical outcome (immediate and long-term). Since there is no current gold standard for a clinical decision, *CEDAR *will contribute to an outcome-oriented conceptualisation of CDM quality: Clinical decisions of a "good quality" are those with a strong association with good clinical outcome.

### Apply methodology to explain variations of care resulting from clinical decision making

Through a multi-centre prospective observational study, a comprehensive model of CDM will be tested in people with severe mental illness in different countries with different mental health service systems. The objective is to extract best practices of CDM, i.e. to identify structure and process variables with a substantial relation to clinical outcome. Since quality of CDM is just one of many factors impacting upon clinical outcome, possible moderators and mediators of the CDM-outcome relation (e.g. sociodemographic characteristics, clinical variables, therapeutic relationship, satisfaction with involvement) will be comprehensively included in the model. People with SMI will serve as the population for the establishment of best CDM practices. In case differences related to diagnoses should emerge, these practices will be specified for different severe mental disorders (e.g. schizophrenia and depression), and can serve as a model to be transferred to other persistent illnesses.

### Strengthen the clinical governance process for improvements in clinical decision making

Furthermore, by specifying the relationship between CDM and outcome, best practices of clinical decision making in the care for people with severe mental illness will be made available to stakeholders (patients, clinician, health care funders) and clinical governance will be strengthened. This will include the question of whether and to what extent clinical evidence and guidelines are put into practice (in the CDM process) in routine care for people with severe mental illness. This will also include a thorough analysis whether the patient/user perspective is actively integrated in the CDM process in routine care. Thus, the study will contribute to routine mental health care being based on an integration of professional and user perspectives.

The central target will be to provide a differentiated answer to the question, "What amount of patient involvement is most beneficial (i.e. substantially related to patient satisfaction, patient behaviour, and clinical outcome) in what kind of clinical decision?" This will be done on a general level, but also take into account variations in service provision between the participating centres in Germany, UK, Italy, Hungary, Denmark, and Switzerland. This will lead to a set of good practice points which will give guidance on how to improve CDM in the service provision for people with SMI.

### Optimising the delivery of health care and translating the results of clinical research into practice

Communication between clinicians and patients builds the context for the delivery of mental health care in the form of specific treatments at the patient-level. While being affected by a wider background of system level variables (i.e. the extent to which local mental health policy and service provider organisations adequately support the provision of evidence-based interventions), CDM can be regarded the primary means for translating the results of clinical research into practice.

There is a lack of knowledge on the scope and quality of CDM in the care for people with chronic diseases such as SMI. In addition, a small number of studies investigating the effect of interventions to improve CDM in mental health have yielded mixed results, and particularly hardly any on clinical outcome. A thorough examination of CDM and its outcome in this field via a multi-centre prospective study will help to fill this gap. By identifying elements of best practice CDM (i.e. aspects of CDM with a substantial relation to good treatment outcome), *CEDAR *will provide evidence directly contributing to optimising the delivery of health care to European citizens. Furthermore, *CEDAR *will pave the way for the development of targeted interventions to improve CDM in mental health.

## List of abbreviations

CEDAR: Clinical Decision Making and Outcome in Routine Care for People with Severe Mental Illness (study acronym); CDIS CEDAR: Clinical Decision Making Involvement and Satisfaction Scale (instrument); CDM: Clinical decision making; CDMS CEDAR: Clinical Decision Making Style Scale (instrument); CDRC CEDAR: Clinical Decision Making in Routine Care Scale (instrument); SMI: Severe mental illness; ANOVA: Analysis of Variance

## Competing interests

The authors declare that they have no competing interests.

## Authors' contributions

All authors were responsible for the conception of the project and drafting the first study proposal. BP, MS, SSt, and TB were involved in writing the manuscript, and all authors critically revised and approved the final manuscript.

## Pre-publication history

The pre-publication history for this paper can be accessed here:

http://www.biomedcentral.com/1471-244X/10/90/prepub
